# Downregulation of miR-211-5p Promotes Carboplatin Resistance in Human Retinoblastoma Y79 Cells by Affecting the GDNF–LIF Interaction

**DOI:** 10.3389/fonc.2022.848733

**Published:** 2022-03-02

**Authors:** Ning Ke, Lin Chen, Qing Liu, Haibo Xiong, Xinke Chen, Xiyuan Zhou

**Affiliations:** ^1^ Department of Ophthalmology, Children’s Hospital of Chongqing Medical University, Chongqing, China; ^2^ Department of Ophthalmology, The Second Affiliated Hospital of Chongqing Medical University, Chongqing, China

**Keywords:** retinoblastoma, carboplatin resistance, miR-211-5p, glial cell-derived neurotrophic factor (GDNF), leukemia inhibitory factor (ILF)

## Abstract

**Purpose:**

To investigate the role of the miR-211-5p-GDNF signaling pathway in carboplatin resistance of retinoblastoma Y79 cells and what factors it may be affected by.

**Methods:**

A carboplatin-resistant retinoblastoma cell line (Y79R) was established *in vitro*. RNA-seq and microRNA-seq were constructed between Y79 and Y79R cells. RNA interference, RT-PCR, Western blot (WB), and flow cytometry were used to verify the expression of genes and proteins between the two cell lines. The TargetScan database was used to predict the microRNAs that regulate the target genes. STING sites and Co-Immunoprecipitation (COIP) were used to study protein–protein interactions.

**Results:**

GDNF was speculated to be the top changed gene in the drug resistance in Y79R cell lines. Moreover, the speculation was verified by subsequent RT-PCR and WB results. When the expression of GDNF was knocked down, the IC50 of the Y79R cell line significantly reduced. GDNF was found to be the target gene of miR-211-5p. Downregulation of miR-211-5p promotes carboplatin resistance in human retinoblastoma Y79 cells. MiR-211-5p can regulate the expression of GDNF. Our further research also found that GDNF can bind to LIF which is also a secreted protein.

**Conclusion:**

Our results suggest that downregulation of miR-211-5p promotes carboplatin resistance in human retinoblastoma Y79 cells, and this process can be affected by GDNF–LIF interaction. These results can provide evidence for the reversal of drug resistance of RB.

## Introduction

Retinoblastoma (RB) is the most common intraocular malignancy in children under 5 years of age (1). In addition, the incidence of RB is one in 15,000–20,000 (2). Although the treatment of RB has been improved obviously, the survival rate of patients is still poor. Chemotherapy is currently recognized as the first-line treatment for RB in children. At present, carboplatin constitutes one of the standard chemotherapeutic agents applied for RB (3), but its clinical application is greatly limited due to acquired drug resistance upon the long-term treatment. Although many studies have clarified the molecular mechanisms and signal pathways closely related to the carboplatin resistance of retinoblastoma (4), the mechanisms remain incompletely elucidated and require further investigation.

Now more and more evidence shows that microRNA is not only widely involved in the occurrence, development, recurrence, and metastasis of various tumors but also related to the generation of tumor drug resistance. These studies have pointed out that microRNA is closely related to the invasiveness and drug resistance of tumor cells, and regulation of microRNA can inhibit the drug resistance of tumor stem cells and improve their sensitivity to chemotherapy (5). Although some articles have studied the relationship between microRNA and RB resistance mechanism (6–9), the studies are scattered, and it is not clear whether there are other signaling pathways involved in RB resistance mechanism. MiR-211-5p has been demonstrated to play an important role in several cancer types, including colorectal cancer (10), non-small cell lung cancer (11), hepatocellular carcinoma (12), and renal cell carcinoma (13). However, the biological role of miR-211-5p in retinoblastoma Y79R cells is still unclear.

The glial cell-derived neurotrophic factor (GDNF) is a small protein that potently promotes the survival of many types of neurons. GDNF is overexpressed in glioma cancer (14), lung cancer (15), and pancreatic cancer (16). However, the GDNF expression in RB has not been reported. Pretreatment of glioblastoma cell lines with GDNF conferred chemoresistance to 1,3-bis(2-chloroethyl)-1-nitrosourea (BCNU) (17). In prostate cancer, exposure to GDNF also induced tumor cell resistance to mitoxantrone and docetaxel chemotherapy (18). GDNF stimulates downstream signal transduction pathways, such as AKT and mitogen-activated protein kinase (MAPK)/extracellular signal-regulated kinase (ERK) pathways. These two pathways are important for cell invasion, survival, proliferation, and differentiation (19, 20). However, to the best of our knowledge, the relationship between GDNF and RB resistance mechanism has not been reported. In the present study, to elucidate the chemoresistance mechanism, we constructed a retinoblastoma cell line Y79 which is a drug-resistant cell line, and then this cell line was used for RNA-seq and microRNA-seq. We distinguished any candidate differentially expressed genes (DEGs) between the two lines. Then, RNA sequencing revealed that GDNF was a gene enriched in drug transport with obvious differences. Then, it was found that GDNF was the target gene of miR-211-5p. Currently, the research on the signaling pathway of miR-211-5p-GDNF has only been reported in the congenital gastrointestinal atresia (21). We further explore the role of this signaling pathway in the mechanism of RB resistance and what factors it may be affected by.

## Materials and Methods

### Cell Cultures and Treatments

The human retinoblastoma cell line Y79 was purchased from Shanghai Zhong Qiao Xin Zhou Biotechnology Co., Ltd. (ZQXZ Biotech, Shanghai, China). The cells were cultured in RPMI-1640 medium containing 10% (v/v) heat-inactivated fetal bovine serum (Gibco, Grand Island, USA) , 2 mM L-glutamine, 100 U/ml penicillin, and 100 μg/ml streptomycin (Gibco, Grand Island, USA) in a humidified atmosphere (95% air, 5% CO_2_) at 37°C. The carboplatin-resistant RB Y79 (Y79R) cells were established by intermittently exposing the RB cells to a high concentration of carboplatin (10 μg/ml) (APExBIO, Houston, USA) for 24 h and then with a normal medium. After the surviving cells return to normal growth, the next dosing treatment is performed, and this process is repeated for about 8 months to obtain drug-resistant cell lines.

### Detection of Drug Resistance

Cell Counting Kit-8 (CCK-8) (Dojindo, Kumamoto, Japan) was used to detect drug resistance. Y79R cells were seeded at a density of 3.0 × 10^4^ cells/well with 100 μl of medium in 96-well plates and treated with different concentrations of carboplatin for 72 h. Cells without drug and medium without cells were served as the controls. Then, 10 μl of CCK-8 solution was added to each well and incubated for 4 h at 37°C. GraphPad Prism 7.0 software was used to calculate the half-maximal inhibitory concentrations (IC50).

### RNA-Seq and MicroRNA-Seq Data Analysis and Pathway Enrichment Analysis

Normal cell lines (C1, C2, C3) and drug-resistant cell lines (D1, D2, D3) were chosen for RNA-seq and microRNA-seq. The differential expression analysis was performed using the DESeq2 (v1.4.5) (22) with Q value ≤ 0.05. The Gene Ontology (GO) (http://www.geneontology.org/) (23) and Kyoto Encyclopedia of Genes and Genomes (KEGG) (https://www.kegg.jp/) (24) enrichment analyses of annotated differently expressed genes were performed by Phyper (https://en.wikipedia.org/wiki/Hypergeometric_distribution) based on the hypergeometric test. The significant levels of terms and pathways were corrected by Q value with a rigorous threshold (Q value ≤ 0.05) by Bonferroni (25).

### Real-Time Quantitative Reverse Transcription Polymerase Chain Reaction (Real-Time QRT-PCR) Validation

Total RNA was isolated from cells using RNAiso Plus (TaKaRa, Tokyo, Japan) and was then converted to cDNA using a gDNA Eraser kit (TaKaRa, Tokyo, Japan). RT-qPCR analysis was carried out in triplicate for each sample using SYBR Green Master Mix (TaKaRa, Tokyo, Japan). Reverse transcription and detection primers were purchased from RiboBio (RiboBio, Guangzhou, China). All procedures were performed according to the manufacturer’s instructions.

### Western Blotting

The total protein was extracted by RIPA (Beyotime, Jiangsu, China), isolated on 4%–20% ExpressPlus™ PAGE Gel (GenScript, Shanghai, China). Then, the protein was blotted onto the PVDF membrane (Millipore, Bedford, MA, USA). The primary antibody blocking solution (Beyotime, Jiangsu, China) was used to block for 1 h, then incubated with mouse monoclonal anti-ACTIN (M02014-5, Boster, China), rabbit anti-GDNF (ab176564, Abcam, Cambridge, MA, USA), and Monoclonal Mouse anti-LIF (MAB250-100, R&D Systems, Abingdon, UK) overnight, and the corresponding secondary antibody was incubated for 1 h. Densitometry of the resulting bands was performed using ImageJ 1.8.0 software.

### Transfection and Small RNA Interference of Selected Genes

In order to verify whether the expression of GDNF affects the drug resistance of Y79 cell lines, siRNA was used to interfere with the expression of GDNF in Y79R cell lines. Both small interfering RNA of GDNF and miR-211-5p mimics were purchased from RIBOBIO (Ribobio, Guangzhou, China). Cells (1 × 10^5^) were seeded in a 12-well plate at 37°C, 5% CO_2_, and cultured for 12 h. The riboFECTTM CP kit (Ribobio, Guangzhou, China) was used for transfection.

### Apoptosis Detection

Cells were inoculated with 3 × 10^5^ cells/well into 6-well plates, cultured overnight, and treated with carboplatin for 48 h. In accordance with Annexin V-APC/PI Apoptosis Detection Kit (BioLegend, San Diego, CA, USA) instructions, the cells were collected, successively Annexin V-APC was added, and the PI was incubated for 10 min in the dark, at room temperature. Flow cytometry was used to detect cell apoptosis.

### Analysis of GDNF Binding Target MicroRNA

The TargetScanHuman 7.1 (26) website (http://www.targetscan.org/vert_71/) was used to analyze the GDNF binding target microRNA.

### Protein Interaction Analysis

The STING website (https://string-db.org/cgi/input.pl) was used to predict the protein which interacts with GDNF in the transport pathway. Then, Co-Immunoprecipitation (COIP) was used to verify the proteins.

### Molecular Docking

AutoDock Vina software is used for this molecular docking work. GDNF (PDB ID: 3FUB) and LIF (PDB ID: 1pvH) were downloaded from the PDB database (https://www.rcsb.org/), respectively. The 3D structure of carboplatin (SDF format files) was downloaded from the PubChem website (https://pubchem.ncbi.nlm.nih.gov/). In addition, the protein was treated with PyMOL 2.4, including the removal of ligand molecules, water molecules, and hydrogen atoms. After the protein and small molecule are ready, the protein core is further defined as the center of the docking pocket, and a cube box that can wrap the protein is set up for the docking conformation search of carboplatin. Finally, the prepared files were used for molecular docking by Vina software.

### Statistical Analysis

All experiments were repeated 3 times, and data were expressed as mean ± standard deviation (mean± SD). GraphPad Prism 7.0 software was used for analysis, and the t-test was used for comparison of differences between groups. If the p value is less than 0.05, the difference is statistically significant.

## Results

### Cytotoxicity Test of Drugs

**Figure 1:**
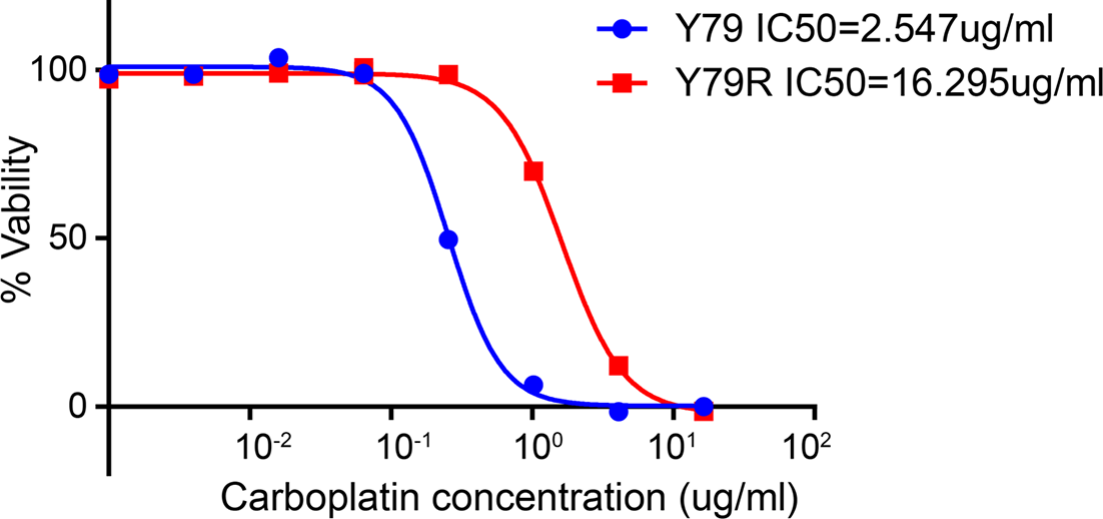
Cytotoxicity of carboplatin in Y79 and Y79R cells. Cell viability was measured by CCK-8 assay. Experiments were done twice in triplicate. Values represent mean ± SD cell viability as percentage of untreated control samples. The half-maximal inhibitory concentrations (IC50) indicate the concentration of a drug required to inhibit 50% cell growth *in vitro*.

After nearly 8 months of inducing resistant cell lines by the high-dose shock method, Y79R cells showed significant resistance to carboplatin, as compared to Y79 cells. The IC50 of carboplatin on the Y79R cell line (16.295 μg/ml) increased 6.4 times compared to the normal culture of Y79 cells (2.547 μg/ml), as shown in **Figure 1**. This finding shows that DEGs needed to be identified to elucidate the intrinsic mechanism of chemoresistance in Y79R cell lines.

### DEGs and Enrichment Analysis of RNA-Seq Data Between Parental Y79 and Y79R Cells

**Figure 2:**
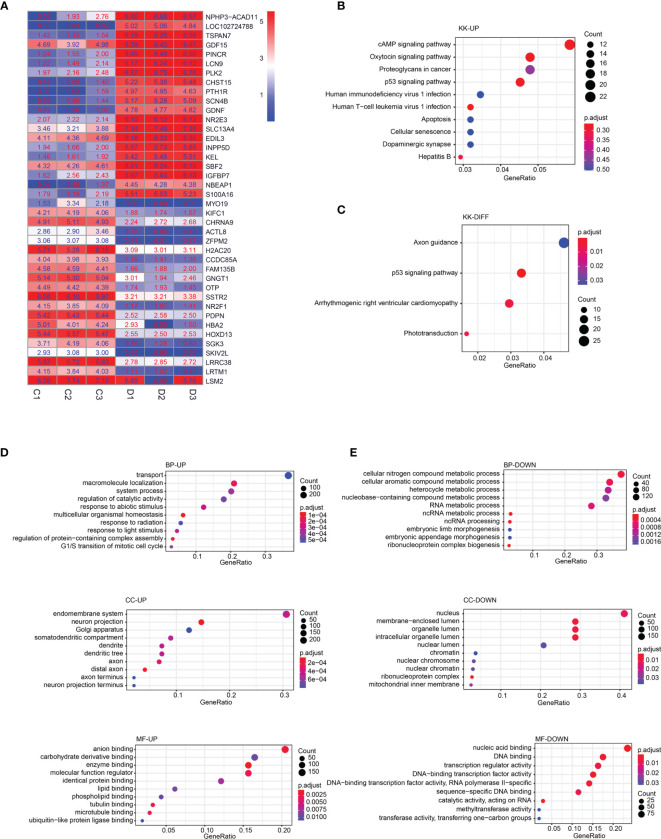
DEGs and enrichment analysis of RNA-Seq data between parental Y79 and Y79R cells. **(A)** Heatmap of 20 upregulated and 20 downregulated genes with top log2FC; FC: fold change. Color indicates the expression level of DEGs with log2(FPKM+1). C:Y79 group; D: Y79R group. **(B)** KEGG pathway enrichment analysis of upregulated DEGs. **(C)** KEGG pathway enrichment analysis of downregulated DEGs. **(D)** GO enrichment analysis of upregulated DEGs. **(E)** GO enrichment analysis of downregulated DEGs (the X-axis is the gene ratio, corresponding to the % column in DAVID’s results table. The Y-axis is the enrichment pathway or GO term. The size of the dot is the number of genes; the color of the dot is the p value. BP, biological process; CC, cellular component; MF, molecular function.

RNA-seq results show that 1,330 differential expression genes are identified; among these genes, 857 genes are upregulated (**Supplementary Table 1**) and 473 genes are downregulated (**Supplementary Table 2**) in Y79R cells compared to normal Y79 cells. The top 20 genes with the largest fold change in the upregulated group and downregulated group are shown in **Figure 2A**. In KEGG pathway analysis, the upregulated genes are enriched in the cAMP signaling pathway, oxytocin signaling pathway, proteoglycans in cancer, and p53 signaling pathway (**Figure 2B**). The downregulated genes have no obvious enrichment pathways, and the total differential genes are enriched in axon guidance, p53 signaling pathway, arrhythmogenic right ventricular cardiomyopathy, and phototransduction (**Figure 2C**). The GO enrichment results shows that in the upregulated group, the biological process (BP) is significantly enriched in transport, the cellular component (CC) is significantly enriched in the endomembrane system, and the molecular function (MF) is significantly enriched in anion binding. Our study suggests that the upregulated genes are significantly enriched in the transport group in BP analysis (**Figure 2D**). In the downregulated group, BP is significantly rich in the cellular nitrogen compound metabolic process, CC is significantly rich in the nucleus, and MF was significantly rich in nuclear acid binding (**Figure 2E**).

### Further Enrichment Analysis of RNA-Seq Data in the Drug Transport Signaling Pathway

**Figure 3:**
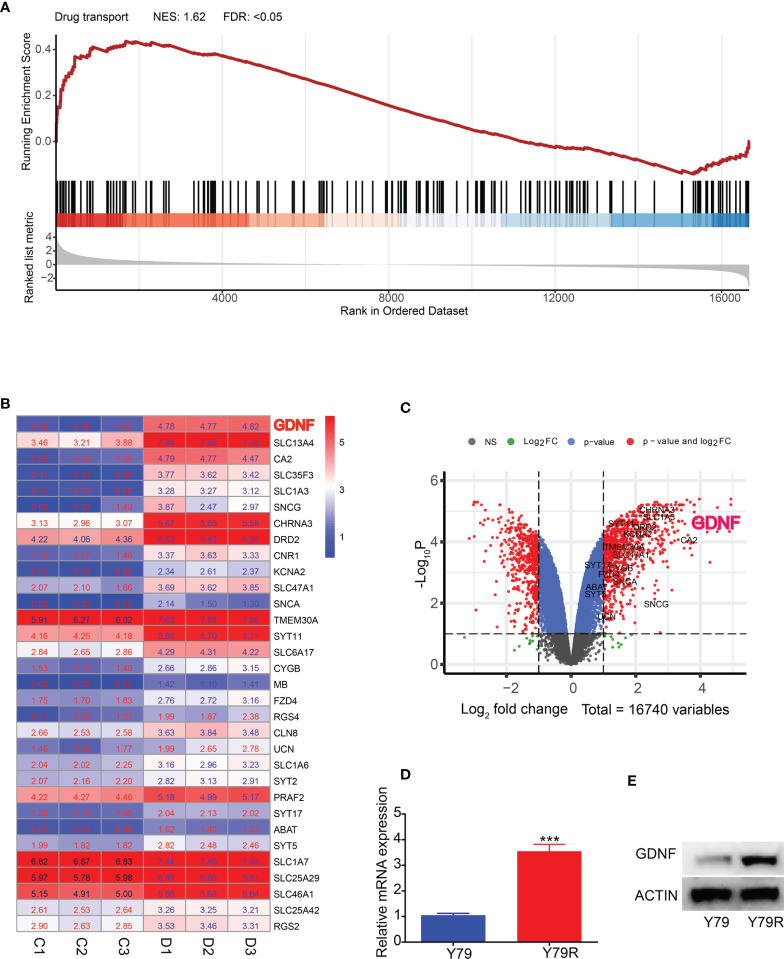
Further enrichment analysis of RNA-Seq data in the drug transport signaling pathway. **(A)** GSEA analysis of drug transport signaling pathway. NES-normalized enrichment score; FDR-false discovery rate. **(B)** Heatmap of DEGs in the drug transport signaling pathway with log2FC. FC, fold change. Color indicated expression level of DEGs with log2(FPKM+1). **(C)** Volcano plot of the distribution of DEGs in the drug transport signaling pathway. NS, No Significant. p: p-value. The red dots indicated DEGs. The other color dots indicated no significantly differential expression. **(D)** QRT-PCR results of GDNF in Y79 and Y79R. **(E)** Western blot results of GDNF in Y79 and Y79R. ^***^Significance at p < 0.001, by t-test between two groups.

We further conducted GSEA analysis and found that drug transport function showed a positive correlation with Y79 drug resistance (**Figure 3A**, **Supplementary Table 3**). Therefore, we decided to further analyze drug transport. We performed a heatmap analysis (**Figure 3B**) and a volcano map analysis (**Figure 3C**) for all genes in drug transport and then selected the gene GDNF, which is the most differentially expressed gene for the next step of verification. RT-PCR results show that GDNF gene expression is significantly increased in drug-resistant cell lines (**Figure 3D**) (p < 0.001), and Western results also showed that GDNF protein was significantly increased in drug-resistant cell lines (**Figure 3E**).

### Effect of GDNF Knocking Down on Drug Resistance of Y79R Cells

**Figure 4:**
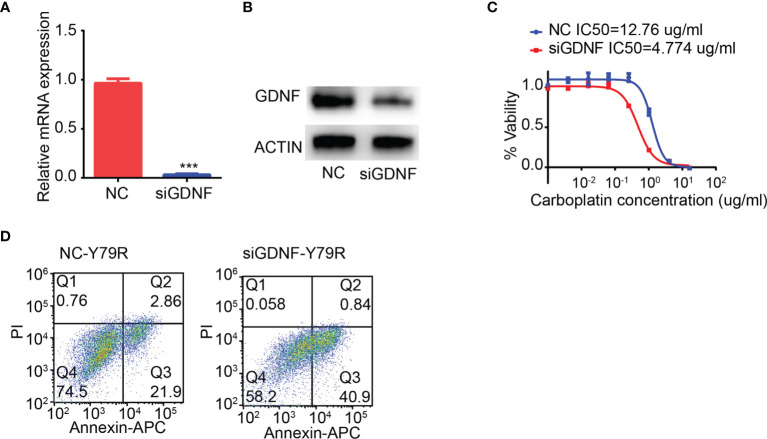
Knocking down GDNF weakens drug resistance of Y79R cells. **(A)** QRT-PCR results of GDNF expression in the Y79R cell line was knocked down by siRNA. **(B)** Western blot results of GDNF in the Y79R cell line was knocked down by siRNA. **(C)** GDNF silencing restored carboplatin sensitivity in Y79R cells. **(D)** GDNF silencing restored carboplatin-induced apoptotic cell death in Y79R cells. ***Significance at p < 0.001, by t-test between two groups.

In order to verify whether the expression of GDNF affects the drug resistance of Y79 cell lines, siRNA was used to interfere with the expression of GDNF in Y79R cell lines. RT-PCR (**Figure 4A**) and WB results (**Figure 4B**) both show that the expression of GDNF in Y79R cell lines is knocked down. The IC50 of Y79R cell lines that interfered with GDNF was significantly lower compared to the control group (**Figure 4C**). Flow cytometric analysis showed that the proportion of apoptotic cells in the interference group is significantly higher than that in the control group (**Figure 4D**). The above results indicated that GDNF knockdown can weaken the drug resistance of Y79R cell lines.

### MiR-211-5p Is Highly Expressed in Drug-Resistant Cell Lines

**Figure 5:**
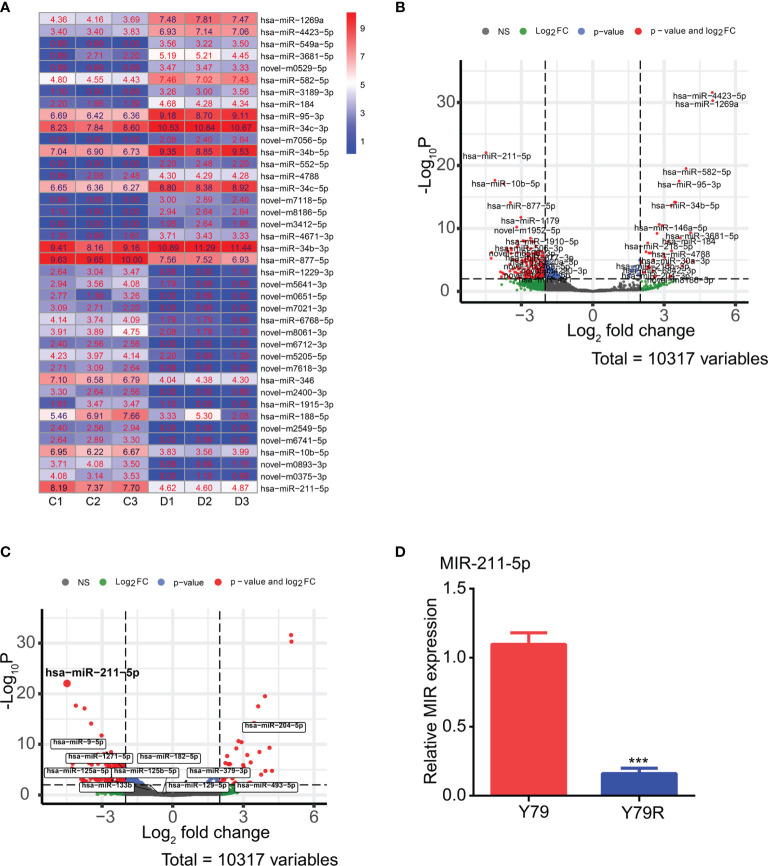
DEG analysis of microRNA-seq data between parental Y79 and Y79R cells and qRT-PCR verification test. **(A)** Heatmap of 20 upregulated and 20 downregulated microRNAs with top log2FC. FC: fold change. Color indicates the expression level of different microRNA expressions with log2(FPKM+1). C:Y79 group, D: Y79R group. **(B)** Volcano plot of differentially expressed microRNAs. NS, No Significant. The red dots indicated different microRNA expressions. The other color dots indicated no significantly differential expression. **(C)** Volcanic map of 34 differentially expressed microRNAs that regulate GDNF. **(D)** QRT-PCR results of MIR-211-5p in Y79 and Y79R. ^***^Significance at p < 0.001, by t-test between two groups.

MicroRNA-seq analysis found that a total of 353 differentially expressed microRNAs were identified in drug-resistant cell lines, of which 55 microRNAs were upregulated (**Supplementary Table 4**) and 298 microRNAs were downregulated (**Supplementary Table 5**). The top 20 differentially expressed microRNAs are displayed by the heatmap (**Figure 5A**) and the volcano map (**Figure 5B**). We analyzed the microRNAs regulating GDNF on the TargetScanHuman 7.1 website, and then we analyzed the intersection between the microRNAs regulating GDNF and differentially expressed microRNAs in the drug-resistant cell line. It is found that there are 34 differentially expressed microRNAs that may regulate GDNF (**Figure 5C**). Among them, miR-211-5p is the most obvious change. Next, qRT-PCR was used to verify the expression of miR-211-5p in drug-resistant cell lines. The results show that miR-211-5p is highly expressed in the Y79R cell line (**Figure 5D**) (p < 0.001).

### Effect of Overexpression of miR-211-5p on Drug Resistance of Y79R Cell Lines

**Figure 6:**
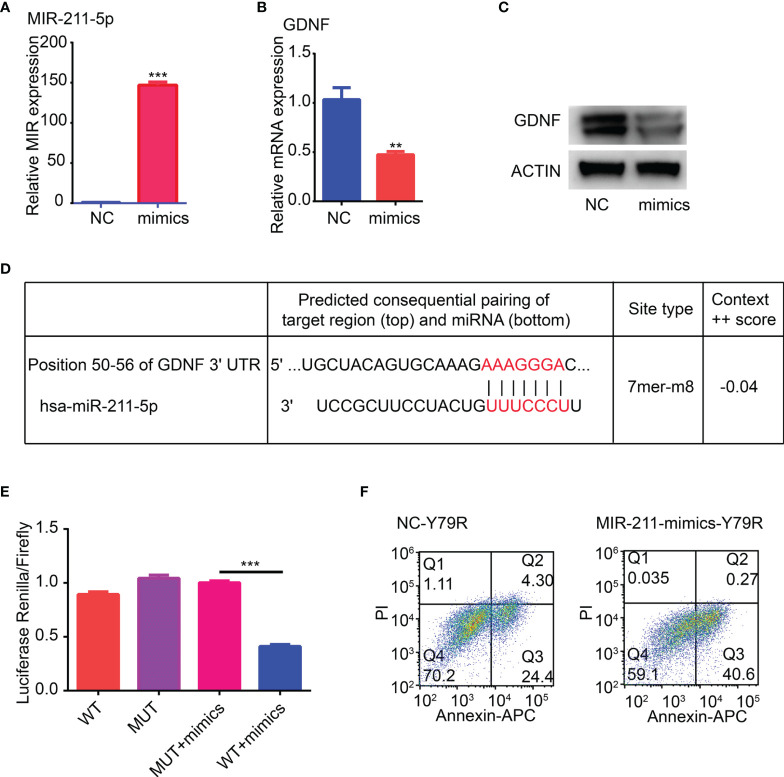
Overexpression of MIR-211-5p weakens drug resistance of Y79R cell lines. **(A)** Overexpression of MIR-211-5p in Y79R. **(B)** QRT-PCR results of GDNF expression in the Y79R cell line overexpressing MIR-211-5p. **(C)** Western blot results of GDNF in the Y79R cell line overexpressing MIR-211-5p. **(D)** Binding target of GDNF and MIR-211-5p was predicted by the TargetScanHuman 7.1 web. **(E)** Luciferase activity was measured using a dual-luciferase reporter gene assay. **(F)** MIR-211-5p overexpression restored carboplatin-induced apoptotic cell death in Y79R cells. **Significance at p < 0.01, ^***^Significance at p < 0.001, by t-test between two groups.

In order to further verify the results of the information analysis, miR-211-5p mimics was used to transfect drug-resistant cell line Y79R. RT-PCR detection found that the RNA expression of miR-211-5p increased 100 times than that in the normal group (**Figure 6A**). The RNA and protein expression of GDNF was significantly reduced in the miR-211-5p overexpression group (**Figures 6B, C**). There is a binding site of miR-211-5p at the 3′UTR end of GDNF by TargetScanHuman 7.1 analysis (**Figure 6D**). Then, we constructed a dual luciferase vector based on the binding site, where WT is a wild-type sequence vector and MUT is a vector that lacks the sequence of the binding site. Then, a dual luciferase experiment was performed. The luciferase activity was significantly lower in the WT group than that in the mutant group (**Figure 6E**) (p < 0.001). Then, the flow cytometric analysis also showed that the proportion of apoptotic cells in the overexpression group was significantly higher than that of the control group (**Figure 6F**). The above results indicate that overexpression of miR-211-5p can reduce the drug resistance of Y79R cell lines.

### Interaction of GDNF With LIF

**Figure 7:**
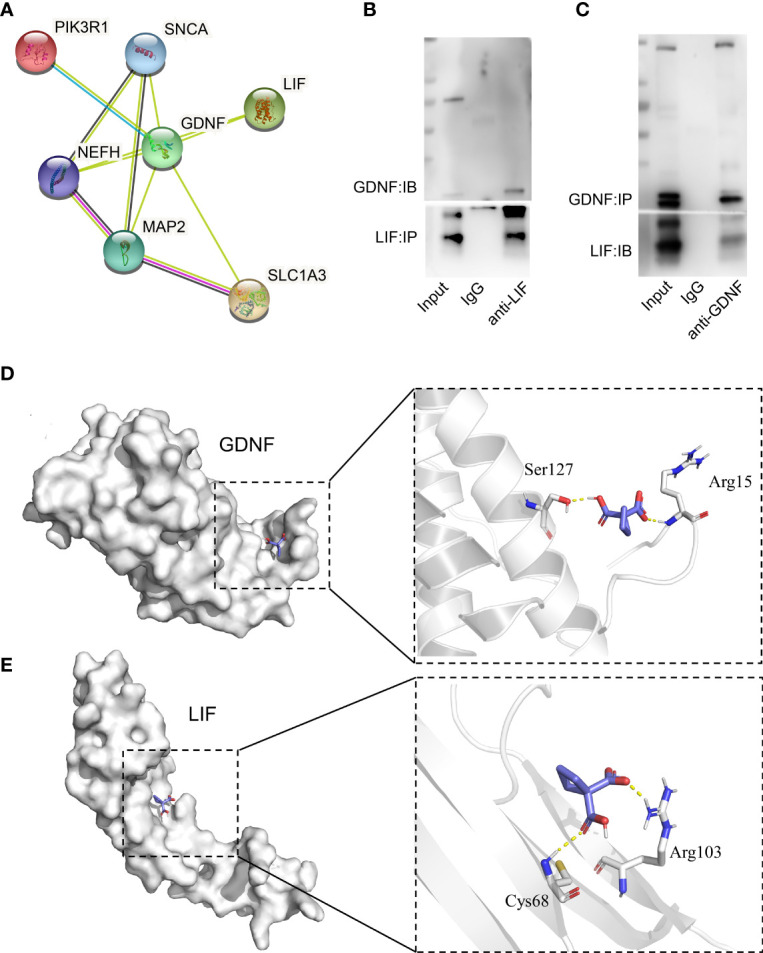
GDNF and LIF interaction. **(A)** The protein interaction with GDNF in DEGs of transport analysis in the sting website. **(B)** Co-Immunoprecipitation analysis between GDNF and LIF (IP : LIF;IB : GDNF). **(C)** Co-immunoprecipitation analysis between GDNF and LIF (IP : GDNF;IB : LIF), **(D)** Vina software predicted the interaction between GDNF and carboplatin. **(E)** Vina software predicted the interaction between LIF and carboplatin.

In order to further study the function of GDNF, we analyzed the interaction of all the proteins in the transport by the String website (http://string-db.org/) and found that GDNF could interact with PTK3R1, NEFH, MAP2, SLC1A3, LIF, and SNCA (**Figure 7A**). In these proteins, only LIF is a secreted protein which can be secreted out of the cell. Therefore, we selected LIF for COIP analysis and found that it interacts with GDNF (**Figures 7B, C**). Then we predicted that GDNF and LIF proteins could interact with carboplatin. The molecular docking results found that both GDNF and LIF could interact with carboplatin (**Figures 7D, E**). Therefore, we speculated that the reason for GDNF promoting carboplatin resistance in Y79R cell lines may be the following: firstly, GDNF interacted with carboplatin. Then, GDNF and LIF are secreted out of the cell, and a large amount of carboplatin is also taken out of the cell, thereby reducing the concentration of carboplatin in the cell to promote drug resistance.

## Discussion

Chemoresistance, either inherent or acquired, is a major constraint of RB treatment. Exploring the mechanisms underlying drug resistance and developing novel therapeutic strategies to overcome such problem are important for RB treatment. Carboplatin is a conventional chemotherapeutic drug that has been used in the past few years for the treatment of RB. Carboplatin is a second-generation platinum compound that can directly inhibit DNA repair to attenuate tumor growth (27); it inhibits tumor growth by binding with DNA and affecting DNA replication. As for the chemoresistance mechanism in RB, proteins such as multidrug resistance-associated proteins (MRP) (28), P-gp (29), and glutathione transferase (30) have been demonstrated to be involved. In the present study, to elucidate the chemoresistance mechanism of carboplatin in RB, we generated transcriptome profiles of Y79R and parental Y79 cells and distinguished any candidate differentially expressed genes (DEGs) between the two lines before performing functional and technical validation studies.

To detect the different expression levels of the gene and protein in the relevant signal pathway between Y79 and Y79R cell lines, the DEGs and enrichment analysis of RNA-Seq data suggested that the upregulated genes were significantly enriched in the transport group in BP analysis in the Y79R cells. The GO enrichment showed that the upregulated genes were significantly enriched in the transport group in BP analysis. In further enrichment analysis of RNA-Seq data in the drug transport signaling pathway, GDNF was a gene enriched in drug transport with obvious differences in Y79R cell lines. As drug transport function showed a positive correlation with drug resistance, we speculated that GDNF is the top changed gene in the drug resistance in Y79R cell lines. The above results indicated that GDNF has a great influence on drug resistance; when GDNF was knocked down, drug resistance decreased in drug-resistant cell lines. In Morandi’s study, GDNF-RET signaling was established as a rational therapeutic target to combat or delay the onset of aromatase inhibitor resistance in breast cancer (31). GDNF confers chemoresistance in a ligand-specific fashion in malignant gliomas (32). Our results are similar to those of the above studies, but the difference is that the sites of influence on the drug resistance mechanism are different, and GDNF acts in cells, while other studies take the next step by acting on receptor RET on the cell membrane (33).

MiR-211-5p functions as a tumor suppressor in hepatocellular carcinoma (34), breast cancer (35), and renal cell carcinoma (36). There are some studies of miR-211-5p on the regulation of tumor drug resistance; miR-211-5p can enable resistance to BRAF inhibitors in melanoma (37). LncRNA KCNQ1OT1 regulates cisplatin resistance in tongue cancer *via* miR-211-5p-mediated Ezrin/Fak/Src signaling (38). Downregulation of circNRIP I suppresses the paclitaxel resistance of ovarian cancer *via* regulating the miR-2 I I-5p/HOXC8 axis (39). However, the biological role of miR-211-5p in retinoblastoma is still unclear. Our results indicate that overexpression of miR-211-5p can weaken the drug resistance of Y79R cell lines. About the signaling pathway involved in miR-211-5p, the miR-211-5p/CENPK axis in tongue squamous cell carcinoma (40) and the miR-211-5pp/BRD4 axis in non-small cell lung cancer (11) have been reported, but not about resistance mechanisms. In our microRNA-seq analysis, miR-211-5p was downregulated in drug-resistant cell lines and directly bound to the 3′ terminal region of GDNF to regulate GDNF degradation. It has been reported that lncrNA-MEG3 has a protective effect on congenital intestinal atretic ganglion cell dysplasia through direct regulation of the Mir-211-5p/GDNF axis, but the role of miR-211-5p/GDNF in carboplatin resistance is still unclear. We further investigated how miR-211-5p regulates GDNF expression upstream. When miR-211-5p was overexpressed, the expression of GDNF decreased significantly. These results can provide evidence for the reversal of drug resistance of RB.

The leukemia inhibitory factor (LIF) is a secreted protein which belongs to the interleukin-6 family of cytokines. LIF has been implicated in many physiological processes including development, hematopoiesis, bone metabolism, and inflammation. Regarding the interaction between GDNF and LIF, the combination of GDNF and LIF could significantly enhance the *in vitro* proliferation of mouse SSCs (41). Upregulation of the receptor components for LIF and GDNF in motoneurons is important for the regeneration of intramuscular motor nerves damaged by muscle contusion (42). LIF may be utilized for signaling mediated by GDNF and may be important in the pathobiology of neuroendocrine tumors (43). We conducted protein interaction analysis and found that there was an interaction between LIF and GDNF, which was consistent with the above research results. The molecular docking results showed that both GDNF and LIF interacted with carboplatin. The cell membrane, cytoplasm, and nuclear protein participate in these resistance mechanisms. Drug resistance at the level of cell membrane reduces drug uptake and increases efflux, leading to a decrease in the absolute concentration of intracellular drugs. For example, P-GP is the earliest ABC transporter discovered, and the high expression of P-GP is also the most classical mechanism of drug resistance (44). Drug resistance at the level of intracellular metabolic processes of drugs strengthens the cell detoxification function, rapidly inactivates the drug, and repairs the DNA damage caused by the drug in tumor cells in time, such as glutathione transferase (GST)-related drug resistance (45). Resistance occurs at the nuclear level such as topoisomerase ii (45). The previous results also showed that the expression of GDNF and LIF significantly increased in the drug-resistant cells (TBALE 1). This supports the hypothesis that the mechanism of GDNF promoting carboplatin resistance might be related to the combination of GDNF and intracellular carboplatin. As GDNF and LIF are secreted into the extracellular environment, a large amount of carboplatin is also taken out of the cell, thus reducing the intracellular concentration of carboplatin and promoting its drug resistance. Nasma D. Eljack’s study supports a major role of passive membrane diffusion in the uptake of cisplatin and suggests that reduced cell uptake is unlikely to be a significant mechanism leading to the development of drug resistance (46). Our results suggested that the resistance of carboplatin was about intracellular metabolic processes of drugs, not at the cell membrane in RB Y79 cells.

**Figure 8:**
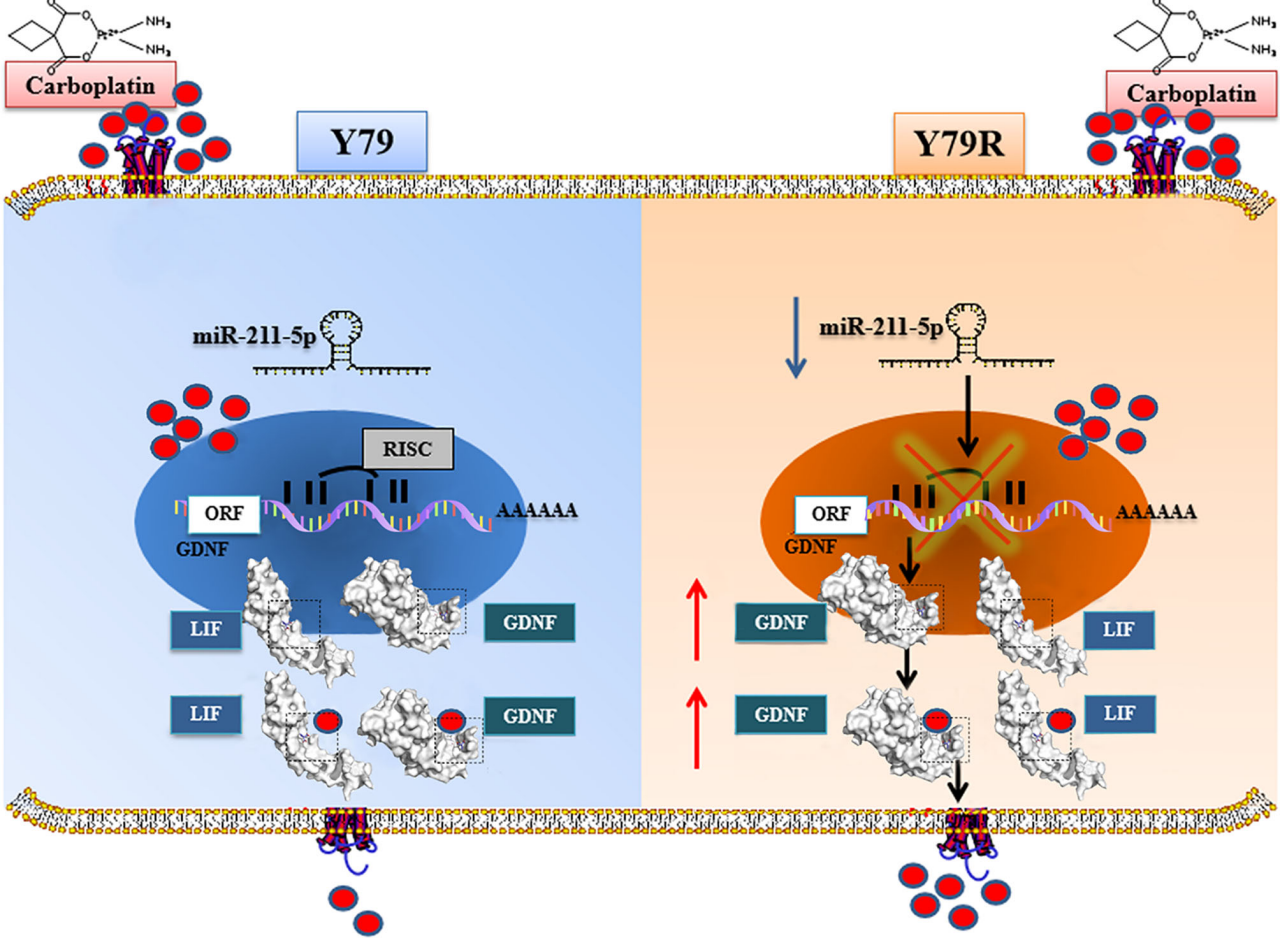
Mechanisms involved in carboplatin resistance in human retinoblastoma Y79R cells. In Y79 cells, downregulation of Mir-211-5p promoted the intracellular expression of GDNF. Highly expressed GDNF binds to more carboplatin and secretes it out of the cell. In addition, GDNF was found to bind to another secreted protein, LIF. It is speculated that LIF can bind carboplatin and excrete carboplatin from cells by secretion. We hypothesize that these processes are involved in cellular drug resistance. ORF, Open Reading Frame; RISC, RNA-induced silencing complex; AAAAAA, 3′polyA tail on behalf of the end of mRNA.

In conclusion, our results suggest that downregulation of miR-211-5p can promote carboplatin resistance in human retinoblastoma Y79 cells, and this process can promote GDNF expression. High expression of GDNF will bind to more carboplatin and secrete it out of the cell. In addition, GDNF was found to bind to another secreted protein LIF. It is also predicted that LIF can combine with carboplatin and take carboplatin out of the cell by secretion. Thus, these events lead to drug resistance of Y79 cells (**Figure 8**). However, whether the actual principle is that this needs further study.

However, some limitations must be addressed. Firstly, we only studied the carboplatin resistance mechanism of Y79, the most common cell line of RB. Whether there are other mechanisms in other cell lines of RB needs to be further studied. Secondly, RNA-seq and microRNA-seq analyses showed that there were many different genes between drug-resistant cells and normal cells, indicating that there were many genes involved in the drug resistance process, and the drug resistance process was a network regulation process in the whole process. In this paper, only drug transport pathways were selected for analysis, and finally, only GDNF with the greatest change in the group was selected for analysis. Few molecules were selected in the experiment, which could not fully reflect the principle of drug resistance. Even for the regulation of GDNF expression, there may be other regulation methods besides microRNAs, and the regulation network of GDNF needs to be further studied and expanded. Finally, this paper only predicted the interaction between GDNF and LIF and carboplatin in the aspect of bioinformatics, which requires further experimental verification.

## Data Availability Statement

The datasets presented in this study can be found in online repositories. The raw rna-seq data generated in this study have been stored in the NCBI Sequence Read Archive (SRA) with Bioproject No.PRJNA796367 (https://www.ncbi.nlm.nih.gov/bioproject/PRJNA796367) and SRA accession number: SRR17567975, SRR17567974, SRR17567971, SRR17567970, SRR17567969, SRR17567968, SRR17567967, SRR17567966, SRR17567965, and SRR17567964 SRR17567973 and SRR17567972.

## Author Contributions

NK and XZ designed the study. NK did the experiments or collected the data for the study. NK, LC, and QL analyzed the data. NK, LC, QL, XC, and HX contributed to writing the paper. All authors contributed to the article and approved the submitted version.

## Funding

This study was supported by the Key Science and Technology Innovation project of Social Undertakings and People’s Livelihood Security in Chongqing (cstc2017shms-zdyfX0021). The funders had no role in the study design, data collection and analysis, decision to publish, or preparation of the manuscript.

## Conflict of Interest

The authors declare that the research was conducted in the absence of any commercial or financial relationships that could be construed as a potential conflict of interest.

## Publisher’s Note

All claims expressed in this article are solely those of the authors and do not necessarily represent those of their affiliated organizations, or those of the publisher, the editors and the reviewers. Any product that may be evaluated in this article, or claim that may be made by its manufacturer, is not guaranteed or endorsed by the publisher.

## Supplementary Material

Supplementary Table 1 857 genes were upregulated.

Supplementary Table 2 473 genes were downregulated.

Supplementary Table 3 All results of GSEA analysis.

Supplementary Table 4 55 microRNAs were up-regulated.

Supplementary Table 5 298 microRNAs were down-regulated.
